# Resin Cement–Zirconia Bond Strengthening by Exposure to Low-Temperature Atmospheric Pressure Multi-Gas Plasma

**DOI:** 10.3390/ma15020631

**Published:** 2022-01-14

**Authors:** Nobuhiro Yoda, Yuri Abe, Yuma Suenaga, Yoshiki Matsudate, Tomohiro Hoshino, Takehiko Sugano, Keisuke Nakamura, Akitoshi Okino, Keiichi Sasaki

**Affiliations:** 1 Division of Advanced Prosthetic Dentistry, Tohoku University Graduate School of Dentistry, Sendai 980-8575, Japan; y.matsudate@gmail.com (Y.M.); takehiko.sugano.p6@dc.tohoku.ac.jp (T.S.); keiichi.sasaki.e6@tohoku.ac.jp (K.S.); 2 FIRST, Tokyo Institute of Technology, Yokohama 226-8502, Japan; yuri_abe@plasma.es.titech.ac.jp (Y.A.); suenaga@plasma.es.titech.ac.jp (Y.S.); aokino@es.titech.ac.jp (A.O.); 3 Joint Research Department of Next-Generation Dental Material Engineering, Tohoku University Graduate School of Dentistry, Sendai 980-8575, Japan; tomohiro.hoshino@gc.dental; 4 Department of Advanced Free Radical Science, Tohoku University Graduate School of Dentistry, Sendai 980-8575, Japan; keisuke.nakamura.e5@tohoku.ac.jp

**Keywords:** low temperature multi-gas plasma treatment, adhesive resin cement, zirconia

## Abstract

The purpose of this study was to investigate the effect of gas species used for low-temperature atmospheric pressure plasma surface treatment, using various gas species and different treatment times, on zirconia surface state and the bond strength between zirconia and dental resin cement. Three groups of zirconia specimens with different surface treatments were prepared as follows: untreated group, alumina sandblasting treatment group, and plasma treatment group. Nitrogen (N_2_), carbon dioxide (CO_2_), oxygen (O_2_), argon (Ar), and air were employed for plasma irradiation. The bond strength between each zirconia specimen and resin cement was compared using a tension test. The effect of the gas species for plasma irradiation on the zirconia surface was investigated using a contact angle meter, an optical interferometer, an X-ray diffractometer, and X-ray photoelectric spectroscopy. Plasma irradiation increased the wettability and decreased the carbon contamination on the zirconia surface, whereas it did not affect the surface topography and crystalline phase. The bond strength varied depending on the gas species and irradiation time. Plasma treatment with N_2_ gas significantly increased bond strength compared to the untreated group and showed a high bond strength equivalent to that of the sandblasting treatment group. The removal of carbon contamination from the zirconia surface and an increase in the percentage of Zr-O_2_ on the zirconia surface by plasma irradiation might increase bond strength.

## 1. Introduction

Zirconia ceramics possess outstanding mechanical properties, such as excellent strength and high biocompatibility for dental applications [1]. When adhering a zirconia crown to a tooth, the preparation of the abutment tooth surface and inner surface of the zirconia crown is essential for proper adherence of the zirconia crown to the abutment tooth by the dental resin cement. Many basic studies have been conducted to evaluate the appropriate treatment methods not only for the inner surface of zirconia crowns but also for abutment tooth surfaces [2]. Among the treatment methods for the inner surface of zirconia, sandblasting with alumina particles is commonly employed, as this procedure cleans the ceramic surface, removes impurities, increases surface roughness, and modifies the surface energy and wettability [3]. However, environmental pollution due to powder scattering when performing sandblasting near a dental chair should be avoided.

Recently, low-temperature plasma jets have attracted attention as effective surface treatment applications in the dental field, especially for zirconia surface treatment. The plasma jet has been found to be effective in decomposing and removing contaminants at the electron level and activating the surface energy [4,5]. Atmospheric moisture is decomposed by high-energy electrons, and OH radicals are produced by plasma generation. As a result, C–C and C–H bonds can be broken and contaminants can be removed from the zirconia surface [6]. These phenomena may increase the surface wettability, which can strengthen the bond strength between zirconia and resin cement [7]. Moreover, low-temperature plasma has been reported to improve the bond strength between zirconia and dental resin cement. [8,9,10,11]. Surface treatment by plasma jet is a clean, environmentally friendly method that does not generate medical waste; thus, it is expected to be used in future dental practice.

The effect of low-temperature atmospheric pressure plasma on surface treatment differs depending on the irradiation conditions. In particular, the type of gas species used to generate plasma has a large effect on the plasma irradiation target [5,6]. The conventional plasma sources are limited in their ability to generate gas species; thus, the previous studies investigated the effect of surface treatment of zirconia using only a few specific gases, including nitrogen, helium, and argon. In addition, the reaction processes and production amounts of reactive species of various gases on the zirconia surface have not yet been verified [11]. In addition, the effect of irradiation time of plasma on zirconia surface treatment remains unclear. Recent developments in low-temperature atmospheric pressure multi-gas plasma jets have been applied to various situations, such as surface treatment [5], treatment of microbial suspensions [12], decomposition [13], hemostasis [14], genome editing [15], and inactivation of oral bacteria [16]. This state-of-the-art plasma equipment can also aid in the surface treatment of zirconia, and the irradiation distance, time, and gas temperature can also be appropriately changed.

This study aimed to investigate the effect of surface treatment using a low-temperature atmospheric pressure multi-gas plasma jet device with various gas species and different treatment times on the bond strength between zirconia and dental resin cement. In addition, the zirconia surface state after plasma treatment was investigated using a contact angle meter, an optical interferometer, an X-ray diffractometer (XRD), and X-ray photoelectron spectroscopy (XPS). The hypothesis of this study was that the gas species used for atmospheric pressure low-temperature plasma jets and the irradiation time would influence the chemical condition of the zirconia surface and the bond strength between zirconia and dental resin cement.

## 2. Materials and Methods

### 2.1. Preparation of Specimens

Zirconia specimens were processed in sections (15 mm × 15 mm × 3 mm) from the discs of 3 mol% yttria-stabilized zirconia (Aadva EI Zirconia Disc, GC, Tokyo, Japan) and polished using a polishing diamond plate with a particle size of 30–40 µm.

**Figure 1:**
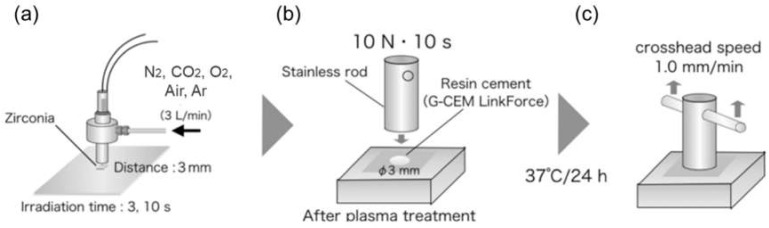
Procedure for evaluating tensile bond strength: (**a**) An atmospheric low-temperature multi-gas plasma jet was used for zirconia surface treatment. (**b**) After zirconia surface treatment, all zirconia specimens were immediately bonded to stainless rods using dental adhesive resin cement. The bond area was circular, with a diameter of 3 mm. (**c**) The bond strength was measured using a universal testing machine.

An atmospheric low-temperature multi-gas plasma jet device [5] was used for zirconia surface treatment. The body of the plasma jet device was grounded and an interior high-voltage electrode was connected to an AC power supply (Plasma Concept Tokyo, Inc.) at 16 kHz and 9 kV. The generated plasma flowed through a 1 mm hole at a flow rate of 3 L min^−1^. Nitrogen (N_2_), carbon dioxide (CO_2_), oxygen (O_2_), argon (Ar), and air were used as plasma gas species. Plasma was irradiated from 3 mm above the zirconia pieces for 3 and 10 s at a room temperature of 20 °C. The cementing area of zirconia surface, which was a circle with a dimeter of 3 mm, was fully treated under this condition of plasma irradiation (Figure 1b). For the alumina sandblasting treatment, 50 µm alumina was used from an injection distance of 5 mm, with an injection time of 5 s and an injection pressure of 0.3 MPa so that the bonding area could be treated sufficiently. Primer containing phosphate monomer, such as 10-methacryloxydecyl dihydrogen phosphate, was not applied to analyze the effect of plasma irradiation alone.

Zirconia specimens were randomly distributed under the following conditions: according to surface treatment: untreated control group, alumina sandblasting treatment group, and plasma treatment group with five different gas species—N_2_, CO_2_, O_2_, Air, and Ar—and two different irradiation times—3 and 10 s for each gas species. As a result, 12 experimental groups were evaluated. The sample size for analysis of variance was calculated using a free software package (G*Power 3.1) [17]. Based on our preliminary study, the following conditions were assumed: alpha = 0.05, power (1-beta) = 0.8, number of groups = 12, and effect size = 0.6. The total sample size was calculated to be 60. Therefore, 5 specimens were prepared for each group.

### 2.2. Evaluation of Tensile Bond Strength between Zirconia and Cement

The procedure for evaluating the tensile bond strength between zirconia and dental adhesive resin cement is shown in Figure 1. After zirconia surface treatment, all zirconia specimens were immediately bonded to stainless rods of 6 mm in diameter and 15 mm in height (SUS303, Stainless Syouji, Tokyo, Japan) using dental adhesive resin cement (G-CEM LinkForce, GC, Tokyo, Japan). Light curing was performed for 10 s using a dental LED light curing unit (G-Light Prima 2 Plus, GC, Tokyo, Japan) under a static load of 10 N in a constant-load device. Excess cement was removed before it was completely cured. Subsequently, the specimens were immersed in water at 37 ± 2 °C for 24 h. Untreated and sandblasted specimens were bonded in the same manner and used as controls. 

The bond strength was measured using a universal testing machine (Autograph AG-I 20 kNT, Shimadzu, Kyoto, Japan) with a crosshead speed of 1.0 mm/min to perform the tensile adhesion strength tests. The maximum load recorded during the test was used to calculate bond strength. Statistical analysis was performed using one-way analysis of variance with surface treatment as a factor. Tukey’s test was used for the post hoc test. All analyses were performed using IBM SPSS Statistics version 22 (IBM Corp., Armonk, NY, USA).

### 2.3. Evaluation of Wettability of Zirconia Surface

A contact angle goniometer (PG-X, MATSUBO, Tokyo, Japan) was used to evaluate the wettability of the zirconia surface treated with sandblasting and plasma irradiation. Three zirconia specimens were separately prepared for each group—untreated, sandblasted, and plasma treated for 10 s with N_2_, CO_2_, O_2_, Air, and Ar gases—using the same device setting described in Section 2.1. In addition, the zirconia specimens after plasma irradiation with N_2_ gas as a representative gas were analyzed for 1, 2, and 3 s to investigate the effect of irradiation time (*n* = 3, for each time). The zirconia specimens treated with sandblasting for 5 and 10 s were also analyzed (*n* = 3, for each time). Therefore, a total of 33 specimens were evaluated. The differences in wettability of the zirconia surface among the groups and irradiation time, in the case of sandblasting or N_2_ gas irradiation, were analyzed using the Kruskal–Wallis test.

### 2.4. Analysis of Zirconia Surface Topography

Surface topography of zirconia specimens treated with sandblasting or plasma irradiation was analyzed using an optical interferometer (TalySurf CCI HD-XL, Taylor Hobson, Leicester, UK) with a 50× objective lens. Three zirconia specimens were separately prepared for each group: control, 5 s sandblasting, and 10 s plasma irradiation with N_2_, CO_2_, O_2_, Air, and Ar gases. Thus, a total of 21 specimens were evaluated. The resolution and the measurement area were set at 2048 × 2048 pixels and 330 × 330 µm, respectively. The acquired images were leveled and processed using a Gaussian filter with a cutoff value of 80 µm to calculate the surface roughness parameters. The surface variation was described using the following three parameters according to guidelines for topographic evaluation [18]: S_a_ (height parameter; arithmetic mean height), S_tr_ (space parameter; texture aspect ratio), and S_dr_ (hybrid parameter; developed interfacial area ratio). The difference in each surface roughness parameter among the groups was analyzed using Tukey’s test.

### 2.5. Analysis of Crystalline Structure of Zirconia Surface

The influence of sandblasting and plasma irradiation using different gas species on crystalline structure of the zirconia specimens was analyzed using a θ–2θ XRD (SmartLab, Rigaku, Tokyo, Japan). The specimens used in the analysis of surface topography were subjected to XRD analysis (i.e., seven groups and *n* = 3 for each group). Diffractograms with Cu-Kα radiation were obtained from 27° to 33° at a scan speed of 10°/min and a step size of 0.02°. The monoclinic volume fraction, Vm, was calculated using the Garvie and Nicholson method modified by Toraya [19,20],

Vm = 1.311 × Xm / (1 + 0.311 × Xm),

with Xm = [Im (−111) + Im (111)] / [Im (−111) + Im (111) + It (101)],

where It and Im represent the integrated intensity of the tetragonal (101) and monoclinic (111) and (−111) peaks. The integrated intensity of each peak was calculated using the device software (PDXL2, Rigaku). The results were analyzed using Tukey’s test.

### 2.6. Analysis of Zirconia Surface Chemical Composition

Surface analysis using XPS (ESCA 1700R, ULVAC-PHI, Kanagawa, Japan) was performed at a tube voltage of 1486.7 eV at 300 W to analyze the change in zirconia surface chemical composition after surface treatment with N_2_ and O_2_ gas as representative gas species. Zirconia specimens were exclusively prepared for this analysis. The zirconia specimens after plasma irradiation for 10 s with both gases using the same device setting described above were used for this evaluation (*n* = 1 for each gas species). 

## 3. Results

### 3.1. Tensile Bond Strength between Zirconia and Cement

**Figure 2:**
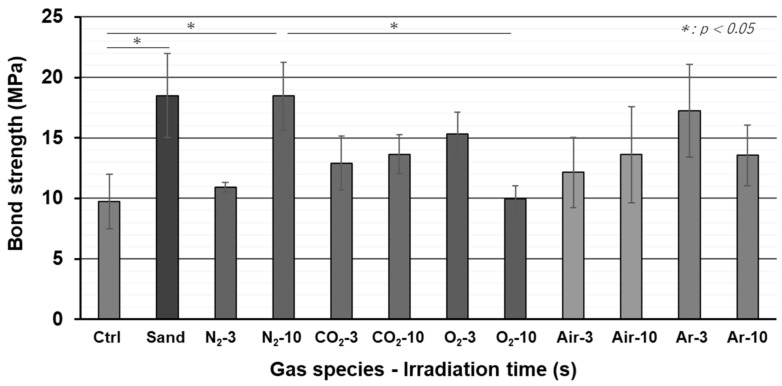
Tensile bond strength between zirconia and resin cement.

The bond strength between zirconia and resin adhesive cement for each surface treatment is shown in Figure 2. Adhesive failure was confirmed at the interface of zirconia and cement in all specimens. Sandblasting treatment significantly increased bond strength. The bond strength after plasma treatment with N_2_ gas for 10 s was significantly increased compared to untreated specimens and showed a high bond strength, equivalent to that after sandblasting treatment. Although no significant differences were observed, bond strength changed depending on the other gas species as well as the irradiation time. 

### 3.2. Water Contact Angle of Zirconia Surface

**Figure 3:**
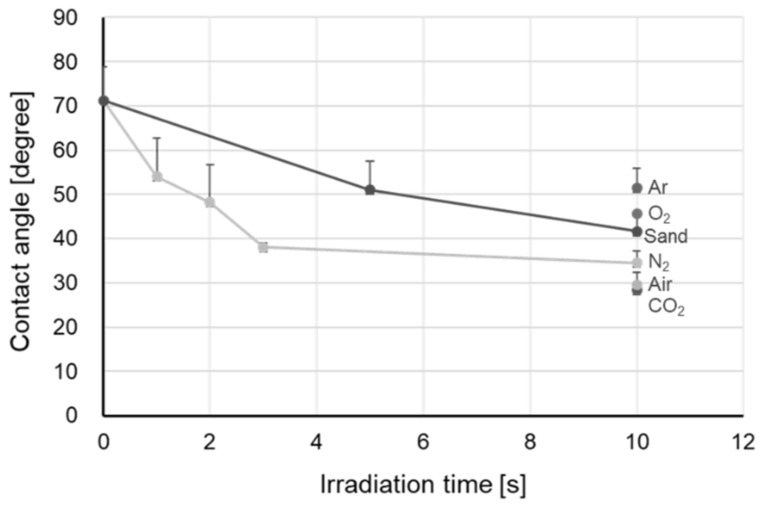
Relationship between plasma irradiation time and contact angle.

The contact angle of the zirconia specimens after plasma treatment was smaller than that of the untreated specimens. In addition, the contact angle varied with the gas species used for plasma irradiation, though the differences between the different gas species were not significant. The highest wettability was demonstrated on zirconia treated by plasma irradiation with CO_2_ gas (Figure 3). 

For both sandblasting and plasma treatment with N_2_ gas, as the irradiation time increased, the contact angle decreased. The significant decrease in contact angle for the 10 s treatment was observed compared to the untreated control sample in both sandblasting and plasma irradiation with N_2_ gas. 

### 3.3. Zirconia Surface Topography 

**Figure 4:**
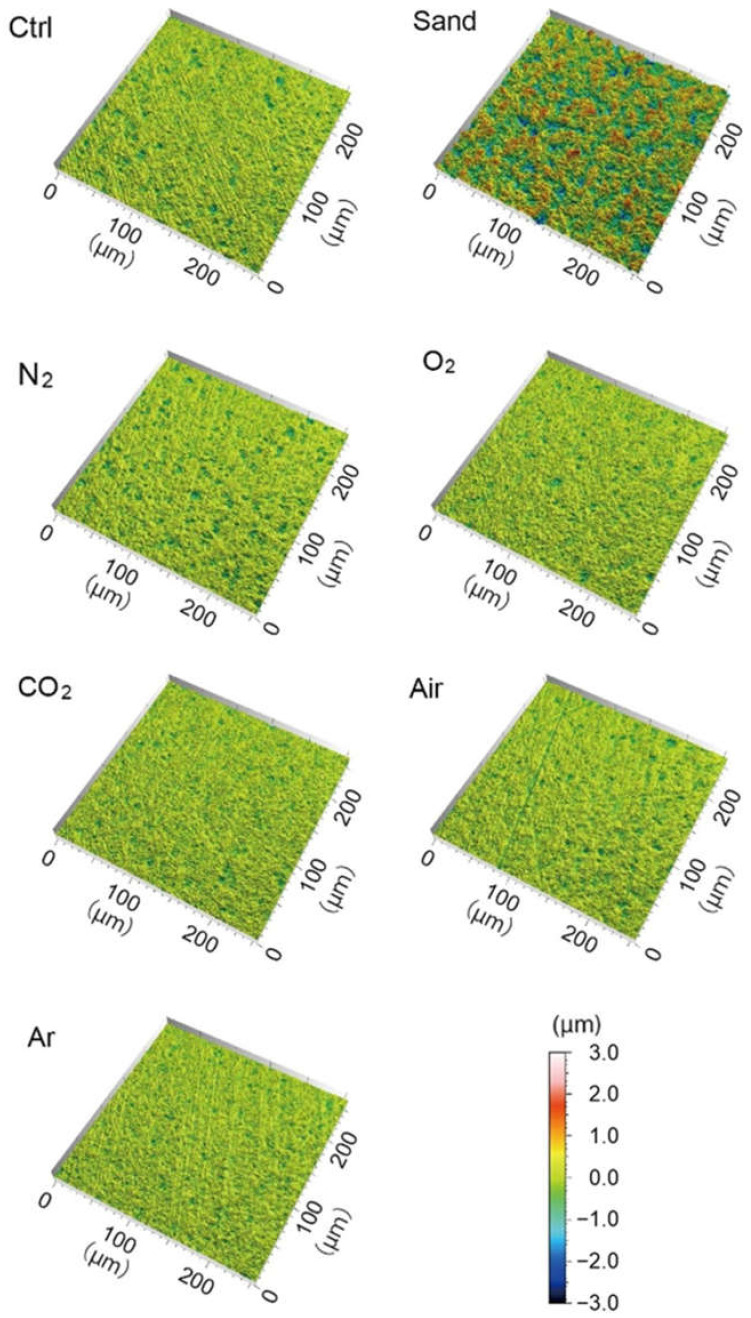
Representative images of zirconia surface after sandblasting or plasma irradiation using different gas species.

Optical interferometry revealed that plasma irradiation did not affect the surface topography of zirconia, irrespective of gas species used for the treatment, whereas sandblasting increased surface roughness (Figure 4). The surface roughness parameters are presented in Table 1. The S_a_ values for the specimens treated with plasma irradiation using different gas species were in the range between 0.1050 and 0.1140 µm, which did not significantly differ from that of control group (0.1123 µm). In contrast, sandblasting significantly increased S_a_ to 0.4713 µm compared to the control group. In association with S_a_ values, S_dr_ was significantly changed by sandblasting. Regarding S_tr_, the values were in the range between 0.839 and 0.925 and there was no significant difference between the groups, indicating that the specimens in each group possessed a spatially isotropic texture.

### 3.4. Crystalline Structure of Zirconia Surface

**Figure 5:**
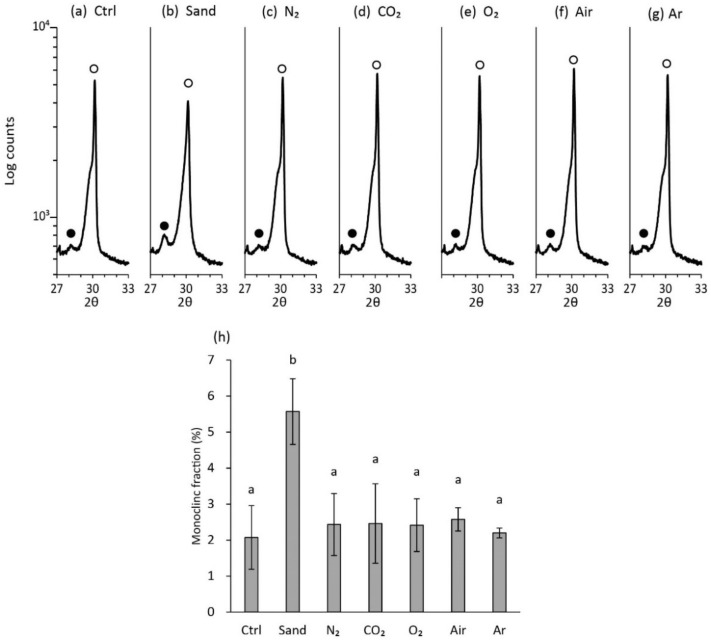
Monoclinic fraction (%) in zirconia specimens treated with or without sandblasting or plasma irradiation using different gas species. The fraction was calculated based on the X-ray diffraction patterns obtained in the 2θ region between 27° and 33°. In (**a**–**g**), black and white circles indicate Im (111) and It (101), respectively. Im (−111) was not detected because of the low monoclinic fraction. Quantitative results are summarized in (**h**). Different letters above columns (a and b) denote significant differences (*p* < 0.01) between the treatment groups.

Representative diffractograms are presented in Figure 5. As all specimens were prepared using a diamond polishing plate with a particle size of 30–40 µm, monoclinic peaks were detected, even in the control group. Quantitative analysis revealed that the monoclinic fractions in the specimens treated with plasma irradiation were approximately 2%, irrespective of gas species used for the treatment, which were almost equal to the monoclinic fraction of control group (2.1%) (Figure 5). Sandblasting resulted in the highest monoclinic fraction of 5.6%, which was significantly higher than those of the other groups.

### 3.5. Zirconia Surface Chemical Composition

**Figure 6:**
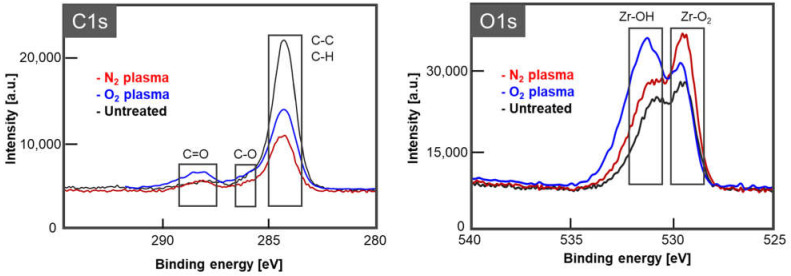
Zirconia surface state after plasma treatment with N_2_ and O_2_ gases: XPS spectra of C1s and O1s. XPS: X-ray photoelectron spectroscopy; Zr: Zirconia.

Figure 6 shows the detailed XPS spectra of the C1s and O1s electron energy levels on the zirconia surface after plasma treatment with N_2_ and O_2_ gases. Carbon contamination on the zirconia surface was removed after plasma irradiation. In addition, the Zr-O_2_ peak strength (approximately 530 eV) and the Zr-OH peak strength (531.6 eV) were found to differ after N_2_ and O_2_ plasma treatment.

## 4. Discussion

The low-temperature atmospheric pressure multi-gas plasma jet device used in this study was able to generate stable plasma using various gas species at low temperatures. A previous study using this device clarified the influence of gas species on the number of reactive species and the reaction process at the gas-liquid interface [6]. A high antimicrobial effect has also been reported; plasma irradiation with CO_2_ and N_2_ gases, which generated the greatest amount of ^1^O_2_ (singlet oxygen) compared to other gas plasmas, killed general bacteria and some fungi [12]. This study shows that this device is useful for the surface treatment of zirconia. Therefore, it might be worth applying to other dental materials.

In context of the effect of plasma irradiation on the bond strength between zirconia surface and dental resin cement, a study by Ito et al. reported that plasma irradiation with helium gas enhanced the bond strength of zirconia to resin cement [8]. In addition, surface treatment of zirconia with non-thermal atmospheric pressure plasma using argon after sandblasting showed a positive effect on the initial shear bond strength [9]. Representative examples of gas species used for plasma discharge in an atmospheric pressure low-temperature plasma treatment apparatus include noble gases, such as helium and argon. This study first explored the effect of plasma irradiation, with various gas species and irradiation times for the surface treatment of zirconia, on the bond strength of zirconia to resin cement. It is valuable to investigate the effect of common gases, not only rare gases, such as helium, because of their cost-effectiveness for future chairside applications.

Plasma irradiation affected the zirconia surface at the electron level via the reaction of ionized and plasmatized gas [8]. It is known that atmospheric moisture is decomposed by high-energy electrons, and OH radicals are produced by plasma generation [6,21]. OH radicals, which possess a redox potential that is higher than those of O_3_, H_2_O_2_, and HClO [22], oxidize organic contaminants at high rate constants (e.g., 10^6^–10^9^ M^−1^ s^−1^) [23]. Therefore, they are capable of degrading various types of organic contaminants. This property of OH radicals, namely, the advanced oxidation process, has been widely applied to the decontamination of pollutants in water and soil [22,23,24]. In the present study, it was revealed that O_2_ and N_2_ plasma irradiation decreased the peaks of C–C and C–H bonds, suggesting that organic contaminants accumulated on the zirconia surface were decomposed by OH radicals generated via plasma irradiation. This finding was in accordance with recent studies in which low-pressure plasma treatment of polymer material and titanium alloy decreased the amount of carbon on the material surface [25,26]. These phenomena might improve the surface wettability [25,26]. An increase in the wettability of the zirconia surface after plasma irradiation, regardless of the gas species, indicates an increase in the zirconia surface energy [11]. A study by Takamatsu et al. investigated the influence of gas species on the reactive species and demonstrated that nitrogen plasma produced higher concentrations of H and OH radicals than other gas species. In addition, high concentrations of ^1^O_2_ were detected in CO_2_ plasmas [6]. These reactive species are considered to act on the surface, improving wettability and contributing to the increase in surface free energy. 

The results of this study indicate that plasma irradiation with N_2_ gas for 10 s was effective in improving the bond strength between zirconia and dental resin cement. It was also demonstrated that bond strength varied depending on the gas species used for plasma generation. Surface analysis by XPS showed that carbon contamination was removed by plasma irradiation, possibly because of the generation of OH radicals, as discussed above. This might contribute to the improvement in bond strength. Comparing N_2_ and O_2_ plasma irradiations, the former resulted in smaller peaks of C–C and C–H bonds than the latter. This may be explained by the difference in the yields of the OH radicals. A previous study demonstrated that N_2_ plasma generated larger amounts of OH radicals than O_2_ plasma [6]. Thus, it is reasonable to assume that N_2_ plasma irradiation resulted in lower contaminants and higher bonding strength. According to optical interferometry, the surface topography of zirconia was not influenced by treatment with plasma irradiation, irrespective of gas species. This suggests that the improvement in bond strength is irrelevant to the increase in surface roughness, unlike sandblasting. O_2_ plasma irradiation did not improve bond strength, although the organic contaminants on the zirconia surface were reduced and the proportion of Zr-OH increased, which plays an important role in chemical bonding between the zirconia surface and resin cement [27]. The data obtained in this study cannot clarify the reason why O_2_ plasma did not improve bond strength. However, it can be speculated that O_2_ and/or superoxide anion radicals derived from O_2_ plasma irradiation can penetrate into the microdefects of the material and subsequently dissipate as a gas during the polymerization of resin cement [27]. O_2_ gas might increase the porosity of the resin cement, weakening the bond strength. Indeed, a study by Liu et al. [28] demonstrated that zirconia surfaces treated with O_2_ plasma showed lower bond strength of porcelain veneers and were characterized by more pores at the interface of the materials than the surface without O_2_ plasma irradiation. In addition, O_2_ inhibits free radical-mediated polymerization of resin cements because it easily reacts with propagating radicals (i.e., free radicals in methacrylate groups generated by initiators), thus terminating the chain reaction [29,30]. Thus, in this study, the longer treatment times (10 s) with O_2_ plasma irradiation might generate more O_2_ gas than shorter treatments (3 s), with the former resulting in lower bond strength. 

Although no significant difference was observed, Ar plasma irradiation increased bond strength. Surface treatment by Ar plasma irradiation might involve several reactive species, such as OH radicals, H radicals, atomic oxygen, and atomic nitrogen, generated via the reaction of semi-stable Ar with oxygen molecules, nitrogen molecules, and water molecules in air [6]. These reactive species might contribute to the removal of surface contamination, and thus, to an increase in bond strength. However, the yield of reactive species generated by Ar plasma irradiation was lower than those generated by O_2_ and N_2_ plasma irradiations [6]. Thus, it is speculated that the increase in bond strength achieved by Ar plasma irradiation for 3 s might also be associated with another effect, such as activation of surface deposits. The decrease in bond strength at the extended irradiation time (10 s) of Ar plasma might be related to an accumulation of oxygen on the zirconia surface layer, as in the case of O_2_ plasma irradiation. However, as Ar plasma irradiation would likely involve oxygen in the reaction at a lower level than O_2_ plasma irradiation, the decrease in bond strength by Ar plasma irradiation for 10 s was lower than that caused by O_2_ plasma irradiation for 10 s. Further studies are needed to clarify both positive and negative effects of Ar plasma irradiation on bond strength.

The wettability of the zirconia surface after sandblasting was increased, which was comparable to a previous similar study [31]. Although the wettability of the zirconia surface was maximized when plasma irradiation was performed using CO_2_ gas, the bond strength was not maximized when CO_2_ gas was used. In addition, although the wettabilities of the zirconia surface in the case of plasma irradiation with N_2_ gas for 3 s and 10 s were similar, the bond strength of N_2_ for 10 s was much higher than that for 3 s. As described above, it is considered that the bond strength between zirconia and resin cement can be affected not only by wettability but also by a particular influence on surface characteristics due to plasma irradiation. It is thus worthwhile to further investigate the influence of different gas species and irradiation time on zirconia surface treatment. Previous studies have revealed that several reactive species other than OH radicals were generated depending on the gas species, and the number of reactive species also changed [6]. The role of these reactive species in association with that of OH radicals should also be further studied. In addition, a plasma jet that can change the gas temperature of plasma was developed, and it has been reported that gas temperature affects the reactive species produced by the plasma [32,33]. Various other parameters, such as gas irradiation time, gas temperature, and gas irradiation distance, should be considered for improving bond strength. By applying the plasma jet device used in this study, it is possible to determine optimal plasma irradiation conditions to improve the bond strength between zirconia and dental resin cement. 

The dedicated primer containing phosphate monomer was not used in this study in order to emphasize the effect of plasma treatment on bond strength. A previous study reported that surface treatment with Ar gas plasma irradiation followed by the application of primer seemed to have limited effect on bond strength [9]. Therefore, further investigation is necessary to analyze how the gas species used for plasma treatment influences bond strength when followed by primer application. In addition, a previous study reported that a higher surface energy value was demonstrated with non-thermal atmospheric pressure plasma irradiation compared to sandblasting [10]. Therefore, further investigation of the influence of the different gas species and time on the water contact angle, especially comparing to the that of sandblasting, would be valuable. 

The present study demonstrated that N_2_ plasma irradiation achieved as high a bond strength as sandblasting. In addition, according to XRD analysis, the former did not induce phase transformation of zirconia, whereas the latter generated a monoclinic phase that may cause an unexpected degradation of material, as in the case of low-temperature degradation [34]. Considering the other potential drawback of alumina sandblasting as mentioned earlier, N_2_ plasma irradiation is thought to have sufficient value for future clinical applications. Therefore, an optimal environment-friendly pretreatment system that can potentially be used at the dental chair-side and replace the sandblasting system may be constructed in the future. 

## 5. Conclusions

The effectiveness of atmospheric pressure low-temperature plasma irradiation for zirconia surface treatment was demonstrated. Plasma treatment with N_2_ gas resulted in the highest zirconia–cement bond strength. In addition, it was suggested that the removal of carbon contamination attached to the surface, and an increase in the percentage of Zr-O_2_ in the zirconia surface by plasma irradiation, might increase bond strength.

## Figures and Tables

**Table 1:** Surface roughness parameters of zirconia after sandblasting or plasma irradiation using different gas species (*n* = 3 for each group).

	S_a_ (µm)			S_tr_			S_dr_ (%)		
	Ave	SD	Stats.	Ave	SD	Stats.	Ave	SD	Stats.
Ctrl	0.1123	0.0075	a	0.883	0.067	a	4.55	0.22	a
Sand	0.4713	0.0319	b	0.925	0.015	a	34.97	0.67	b
N_2_	0.1127	0.0051	a	0.916	0.039	a	4.81	0.31	a
CO_2_	0.1110	0.0010	a	0.903	0.008	a	4.92	0.51	a
O_2_	0.1140	0.0040	a	0.904	0.006	a	4.93	0.95	a
Air	0.1050	0.0036	a	0.856	0.054	a	4.73	0.25	a
Ar	0.1140	0.0020	a	0.839	0.154	a	4.75	0.66	a

Different letters in columns for each parameter denote significant differences (*p* < 0.01) between the treatment groups.

## Data Availability

The data presented in this study are available on request from the corresponding author.
